# The effect of social norms on vegetarian choices is moderated by intentions to follow a vegetarian diet in the future: Evidence from a laboratory and field study

**DOI:** 10.3389/fpsyg.2023.1081700

**Published:** 2023-03-08

**Authors:** Alya Hammami, Armelle Garcia, Nicolas Darcel, Suzanne Higgs, Olga Davidenko

**Affiliations:** ^1^Université Paris-Saclay, AgroParisTech, INRAE, UMR PNCA, Palaiseau, France; ^2^School of Psychology, University of Birmingham, Birmingham, United Kingdom

**Keywords:** food behavior change, vegetarian choices, social norms, intentions, moderator

## Abstract

Social norms could be a tool in dietary transition toward more sustainable diets, but the results of social norms interventions aimed at encouraging the selection of plant-based foods to date have been inconsistent. One reason for this might be because there are important moderating factors that have yet to be investigated. Here we examine social modeling of vegetarian food choices and test whether modeling is dependent upon individual intentions to follow a vegetarian diet in the future in two different settings. In a laboratory study of 37 women, participants with low intentions to become a vegetarian consumed fewer plant-based foods in the presence of a vegetarian confederate, compared to eating alone. In an observational study of 1,037 patrons of a workplace restaurant, participants with a higher score of on vegetarian intentions had a greater likelihood of taking a vegetarian main course or starter, and a vegetarian social norm was associated with a greater likelihood of a vegetarian choice for the main course but not for the starter. These data suggest that participants with low intentions to follow a vegetarian diet may exhibit reactance against an explicit vegetarian norm in an unfamiliar context (as in Study 1) but that general norm following regardless of dietary intentions be more likely when it is conveyed implicitly in a familiar context (as in Study 2).

## Introduction

A major feature of recent policy initiatives aimed at tackling the climate emergency and achieving sustainable development goals is the promotion of healthy and sustainable diets (Ipcc, 2022). Given that livestock production is estimated to be responsible for up to 14% of the global greenhouse-gas emissions that contribute to climate change (Gerber et al., 2013) and consumption of high amounts of red and processed meat is also associated with health risks (Papier et al., 2021), one way of achieving health and environmental gains is to encourage a shift from diets high in animal-based protein sources to diets that are more plant-based (Springmann et al., 2018). According to the EAT-Lancet Commission on Food, Planet, and Health report, a healthy and sustainable diet should have no more than 26 kg of meat and fish per year (Willett et al., 2019). Yet, meat consumption in many countries far exceeds this recommendation (Godfray et al., 2018; Stewart et al., 2021). While consumption of plant-based or vegetarian meals has increased in recent years in countries such as the United Kingdom (Alae-Carew et al., 2022), evidence suggests that for many consumers, willingness to reduce or replace meat in the diet is low (Macdiarmid et al., 2016). Greater understanding of the factors that influence selection of plant-based foods will assist in the development of new approaches to promoting the transition to more healthy and sustainable diets.

There are many reasons why some people find it difficult or may be resistant to switching to a more plant-based diet (Zur and Klöckner, 2014). Meat is a source of high-quality protein and perceptions about the benefits to health of consuming meat reduces willingness to substitute meat with alternatives (Hartmann and Siegrist, 2020). Consumers may also be unwilling to substitute meat for plant-based alternatives because they find eating meat is pleasurable and consider it to be a traditional part of the diet (a meal is not complete without meat) (Macdiarmid et al., 2016). Meat also has significant symbolic and social value: it is associated with power, status and wealth (Fiddes, 2004; Rothgerber, 2013) and reducing meat intake may be seen as challenge to social status and identity (Kildal and Syse, 2017). In addition, a lack of support from family and friends and perceived social prejudice toward people who follow plant-based diets have been found to be a barrier to dietary change (Graça et al., 2019). Because eating meat is considered to be a normal dietary habit for many consumers, approaches to promoting consumption of plant-based alternatives will need to consider norms around meat consumption (Horgan et al., 2019).

Descriptive social eating norms are perceived standards for what constitutes appropriate consumption, whether that be amounts of foods or specific food choices, for members of a social group. Descriptive social eating norms may be communicated directly *via* cultural practices and rules (e.g., beliefs about what constitutes a proper meal) or by the behavior of group members in a given situation (e.g., food choices of peers) (Higgs, 2016). People are inclined to follow descriptive social eating norms and this tendency to adapt one’s behavior to be similar to that of other people is known as modeling (Vartanian et al., 2013). According to several reviews and meta-analyses, there is strong evidence for socially normative modeling of food choice and intake (Robinson et al., 2014; Cruwys et al., 2015; Vartanian, 2015). Direct modeling of choice of meat versus vegetarian options has been observed in a restaurant setting: Christie and Chen (2018) reported that patrons were more likely to choose a meat (vegetarian) main course if the person ahead of them in the lunch queue chose a meat (vegetarian) option. It has also been reported that meat-eaters who are accompanied by vegetarians are more likely to choose a vegetarian dish than are meat-eaters who are accompanied by other meat-eaters and the likelihood of choosing a vegetarian meal increases as the number of vegetarian co-eaters increases (Einhorn, 2020). The powerful effect of social eating norms helps to explain why individuals whose social/family group eats a lot of meat might find it difficult to reduce meat intake (Lea and Worsley, 2003), because such behavior would go against the prevailing social norm. However, social norms also offer an opportunity for changing behavior *via* the use of norm-based interventions.

There is accumulating evidence that descriptive social norm-based messaging can be an effective approach to change food consumption (Higgs et al., 2019). For example, presenting information that most fellow diners at a restaurant purchased vegetables with a meal was associated with an increase in purchase of meals with vegetables (Thomas et al., 2017; Collins et al., 2019). Norm-based messages have also been trialed to promote orders of meatless meals in a café (Sparkman and Walton, 2017). Customers waiting in line for service who saw information about a growing number of Americans who are trying to eat less meat were twice as likely to order a vegetarian lunch compared to a control group who saw information about Americans trying to limit social media use. These data suggest that descriptive social norms could be harnessed to assist in dietary transition toward more sustainable diets. However, more recent studies have highlighted that social norm interventions focused on promoting the consumption of vegetarian meals are not always successful (Sparkman et al., 2020; Aldoh et al., 2021; Alblas et al., 2022; Çoker et al., 2022). One reason for this may be that the intervention is only effective for certain subgroups of the population, which leads to underestimating the size of the effect when considering the whole population. It is therefore important to establish which factors might moderate the effects of descriptive social norms on vegetarian food choices because this might allow better targeting of interventions to specific populations in future.

It has been theorized that the response to social norms is moderated by the level of engagement with and attitude toward the normative behavior (Lapinski and Rimal, 2005). Having a less positive attitude toward and not identifying strongly with the social norm is associated with a stronger response (greater modeling) because under these circumstances, people are open to being influenced by accessible and salient information that the norm provides (Lapinski et al., 2017; Cialdini and Jacobson, 2021). This might be one reason why a social norms message aimed at increasing consumption of fruit and vegetables was effective in participants who had low levels of habitual vegetable intake but had no effect on high consumers (Robinson et al., 2014). Omnivores vary in their attitudes toward vegetarianism with some being open considering adopting a vegetarian diet but others having very little intention to adopt a vegetarian diet in the future. It might be predicted that individuals who have a strong intention of adopting a vegetarian diet in the future might be less susceptible to social norms than individuals with a weaker intention because the latter will identify with the behavior less strongly and be more influenced by the norm. In support of this prediction, having a strong positive personal norm about meat reduction (i.e., already feeling an obligation to reduce meat intake) weakened the effect of social norms message on intentions to reduce meat intake (de Groot et al., 2021). To the best of our knowledge, no studies to date have examined the moderating influence of dietary intentions. Hence, the novel contribution here is to investigate, for the first time, whether dietary intentions moderate the effect of a descriptive social norm on plant-based food choices. Furthermore, we also examine norm following in both a controlled, laboratory setting (Study 1) and in a natural workplace setting (Study 2) to allow us to establish in Study 1 whether exposure to a social norm has a *causal* effect on vegetarian food choices and in Study 2 whether the effect can also be observed in a natural setting when people are making real life food choices. We predicted that there might be modeling of vegetarian food choices in both situations but that this effect might be stronger in participants with a lower intention to follow a vegetarian diet in the future due to the norm being more salient to them.

## Study 1

### Materials and methods

#### Participants

Forty female participants from the Paris region, aged between 18 and 55 years, were recruited through advertisement using a public online platform (Information Relay in Cognitive Sciences, www.risc.cnrs.fr) completed by a recruitment agency (www.eurosyn.fr). Minimal sample size was estimated based on a similar study conducted on food choices at a buffet (Robinson and Higgs, 2013) and a power analysis that showed that 34 participants were a minimum to detect a medium effect size for a within-subjects ANOVA for main effects and interactions assuming alpha of 0.05 and power of 0.8. We recruited women only to avoid the effects of impression management which have been reported when unfamiliar women eat with men (Vartanian, 2015). To disguise the aims of the study, it was advertised as research examining the appreciation of foods in a buffet. This cover story was used to minimize the effect of demand characteristics, but no deception was involved as the participants were fully informed about what would be asked of them if they agreed to take part in the study. Participants following any diet involving food exclusions (including being flexitarian or vegetarian), suffering from food allergies, avoiding the foods used in the study or scoring above 14 on the restraint scale or above 13 on the disinhibition scale of the Three Factor Eating Questionnaire were not eligible to participate in the study (Lesdéma et al., 2012). Thirty-seven participants were included in the final sample. One participant was excluded because she was not hungry on the day of the experiment, one participant did not attend the first test day, and one participant was excluded after analysis for outliers. Each participant was reimbursed 40 € for taking part to the study. The study was conducted according to the Helsinki declaration guidelines and all procedures were approved by Ethics Committee of Université Paris-Saclay (decision no. 18–533). Written informed consent was obtained from all participants.

#### Design

The study used a within-subject design with two conditions. In the experimental condition, the participant had lunch in presence of a vegetarian confederate (a researcher who was introduced to the participant as a fellow participant) and in the control condition the participant had lunch alone. Participants were invited to take part to both sessions, spaced by 1 week. The order of the conditions was randomized across participants. Rated intention to become vegetarian in the future was included as a moderating factor.

#### Confederate

To avoid the influence of impression management concerns around food choices that are heightened when women eat with men (Vartanian, 2015) the confederate was always a woman. The confederates were instructed to behave similarly for all participants. The confederate was instructed to select her food items before the participant did and to do so in clear view of the participant. While making her choices, the confederate stated that she is a vegetarian. The food choices and quantities consumed by the confederate were pre-determined by the experimenter. There were three confederates, and all received training and checks by the lead researcher, ensuring that they selected the correct types and amount of food during the experimental sessions.

#### Buffet

The meal offered to the participants was a self-service cold buffet in which food items were placed on separate plates arranged on a buffet table. All food was purchased from local supermarkets. The vegetarian option was falafel (15 falafels, 650 g, 211 Kcal/100 g) while the meat option was roasted chicken slices (650 g, 97 kcal/100 g). A salad bar with additional plant-based foods (grated carrots (530 g, 73 kcal/100 g), green salad (160 g, 26 kcal/100 g), pasta salad (640 g, 141 kcal/100 g) and lentil salad (680 g, 142 kcal/100 g)) was also offered. The desserts offered were a brownie (680 g, 430 kcal/100 g), cottage cheese (500 g, 71 kcal/100 g) and seasonal fruit (1,000 g, 60 kcal/100 g). Participant’s food intake was measured by weighing all foods presented in the buffet before and after each session. Participants’ leftovers were weighed as well.

#### Measures

Prior to taking part in the study, the participants completed a screening questionnaire, and only participants passing the inclusion/exclusion criteria were invited to take part in the study. Apart from the inclusion criteria, the screening questionnaire contained questions on participant’s age, weight and height, as well as a food frequency questionnaire (FFQ) for habitual intakes of meat, poultry and legumes. Before and after each experimental session, participants’ baseline hunger, fullness and desire to eat were measured on a 100-cm visual analog scale (VAS). For example, to measure hunger, participants were asked to indicate “how hungry are you right now” between “not at all” and “extremely.” At the end of each experimental session, participants rated their appreciation of the buffet by answering 3 questions (7-point Likert scale) asking about the nutritional quality, variety, and pleasantness of the food offered at the buffet. A fourth question concerned the novelty of the served foods and asked the participants to list foods that were new to them. Intention to follow a vegetarian diet in the future were assessed at the end of the second experimental session using a questionnaire derived from the theory of planned behavior (Povey et al., 2001). Participants were asked to evaluate to what extent they intended to follow a vegetarian diet in the future using a Likert scale (from 1 = “strongly disagree” to 7 = “strongly agree”). Lastly, participants were asked whether they had guessed the aim of the study (“according to you what was the purpose of this experiment?”).

#### Procedure

All experimental sessions took place between 12:00 and 14:00 on weekdays. For each session, the participant was asked to refrain from snacking between her breakfast and the experimental session. Upon arrival on the first test day, the participant was informed about the study details and asked to provide written informed consent. Then she completed the hunger, fullness and thirst ratings. After that, the experimenter invited her to choose from the buffet (either alone or with the confederate depending on condition for that day). The participant was told to serve herself at the buffet with any foods that she preferred to eat and eat as much of those foods as she liked. Immediately after the lunch, the participant was asked to complete the second hunger, fullness and thirst ratings as well as to rate her appreciation of the lunch. The second test day was similar to the first one, except for the addition of the questionnaire on intentions to become vegetarian in the future and the question on the objectives of the study. Participant was then debriefed by the experimenter who asked verbally her if she appreciated the buffet, which foods did she like or dislike, and finally the participant was paid and thanked for her time.

#### Statistical analysis strategy

All statistical analyses were performed using R (version 3.6.3) and R-Studio (version 1.1.463).

We analyzed the effect of vegetarian social norms and dietary intentions on amounts (g) that participants consumed of the following: total intake, falafel intake, chicken intake, and plant-based food intake (i.e.: accumulated amounts of grated carrots, green salad, pasta salad, lentil salad and falafels).

To analyze the effect of the presence of the confederate and any moderation by intention to become vegetarian, we conducted multiple linear regressions for each of the meal items cited above. Main variables of interest were study condition and vegetarian intention. The interaction between these variables also was included. Models were adjusted for food liking and the reported frequency of meat, chicken and falafel, with session order, participant ID and participant’s age considered as random factors. Analysis was conducted after all data were collected.

### Results

#### Participant characteristics and baseline measures

The participants’ mean age was 35.6 years (SD = 9.3). Mean BMI was 21.2 kg.m^−2^ (SD = 1.9). The mean restraint score derived from TFEQ was 4.8 (SD = 5.65), disinhibition score was 3.56 (SD = 4.12) and hunger score was 4.08 (SD = 5.65) suggesting that dieting and overeating tendencies were low in this sample. Mean intention to follow a vegetarian diet in the future was 3.54 (SD = 1.73). Eighty-four percent (31/37) of the participants reported liking falafel and 81% (30/37) of participants reported liking chicken which suggests that both dishes were equally and relatively well appreciated by the participants.

The participants reported consuming on average 0.40 (SD = 0.30) portions of meat, 0.21 (SD = 0.17) portions of poultry and 0.18 (SD = 0.21) portions of legumes per day. Appetite ratings were consistent with the participants being hungry in both experimental conditions as the average hunger score before the buffet when eating alone was 71.6 (SD = 6.1) and 72.5 (SD = 5.34) when eating with a vegetarian confederate (*t*-test: *p* = 0.48). None of the participants guessed the exact purpose of the study.

#### Total food intake

**Figure 1:**
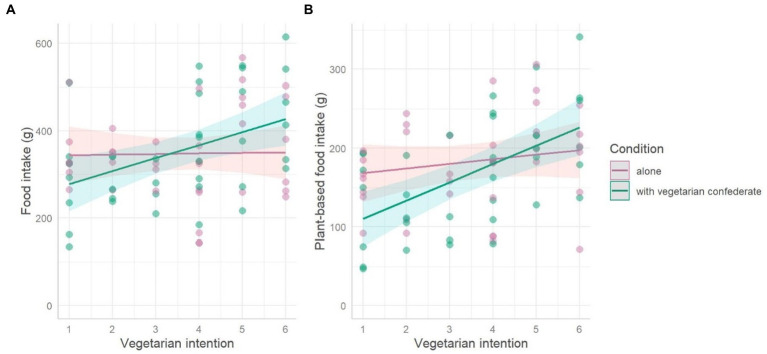
Low intentions to follow a vegetarian diet in the future result in a lower total **(A)** and plant foods **(B)** intakes in presence of a vegetarian confederate. The dots represent the data. The lines represent estimated slopes per study condition. Colored areas represent 95% confidence intervals based on estimated standard errors (+/− 1.96 SE).

There was no effect of condition (*p* = 0.054) nor dietary intention (*p* = 0.896) on total intake. There was a significant interaction between condition and intention on total intake: there was a positive association between vegetarian intentions and total food intake, but only in the presence of the confederate (estimate: 28.4 +/− 11.8 g, T(33.5) = 2.392, *p* = 0.023). Table 1 presents the detailed results and Figure 1A visualizes the data.

**Table 1:** Effect of the presence of a vegetarian confederate and of vegetarian intentions, as well as of their interaction, on intakes at the buffet.

	Estimate (g)	SE (g)	df	*T*-value	*p*-Value
Total					
Condition	−94.3	47.3	33.77	−2.00	0.054
Intention	1.4	10.7	53.29	0.13	0.897
Condition*Intention	28.4	11.9	33.51	2.39	0.023
Plant-based					
Condition	−76.0	24.5	34.34	−3.10	0.004
Intention	5.9	5.9	55.76	1.00	0.323
Condition*Intention	17.5	6.2	34.09	2.84	0.008
Falafel					
Condition	−17.2	11.7	35.28	−1.47	0.152
Intention	0.8	2.5	57.77	0.31	0.760
Condition*Intention	5.3	3.0	35.00	1.80	0.081
Chicken					
Condition	−3.8	9.1	37.75	−0.42	0.678
Intention	−1.6	2.0	67.83	−0.78	0.437
Condition*Intention	0.6	2.3	37.47	0.25	0.802

SE, standard error; df, degrees of freedom.

#### Plant-based food intake

While higher intentions did not have a significant effect on intake (*p* = 0.32), there was an interaction such that there was a positive association between vegetarian intentions and plant-based food intake, but only in the presence of the confederate (estimate: 17.5 +/− 6.2 g, T(34.1) = 2.839, *p* = 0.008). Participants with the lowest intentions ate 76.0 +/− 24.5 g less in the presence of a confederate compared to the control condition (T(34.3) = −3.101, *p* = 0.003). Table 1 presents the detailed results and Figure 1B visualizes the data.

#### Falafel intake

There was no effect of condition (*p* = 0.152) nor dietary intention on falafel intake (*p* = 0.760). There was no significant interaction between condition and dietary intention on falafel intake (*p* = 0.081) (Table 1).

#### Chicken intake

There was no overall effect of condition (*p* = 0.678), nor of dietary intention on chicken intake (*p* = 0.436). There was no interaction between condition and dietary intention on chicken intake (*p* = 0.801) (Table 1).

## Study 2 - social modeling in natural conditions

### Materials and methods

#### Setting

Data collection was conducted in a university restaurant serving the academic staff of a Parisian University. In this restaurant, both vegetarian and meat food options are available daily. Meal prices are identical, regardless of whether the patrons choose a vegetarian or a meat option, and how many components (starter, dessert, and main dish) they choose. This restaurant is organized as a linear buffet such that patrons progress along the buffet and select meal components one after another.

#### Participants

Observations were conducted on patrons having their lunch at the restaurant. For each observed participant, the person immediately ahead in the queue was defined as the source of social norms (referred to as the model hereafter). Patrons who could not be linked to a model (e.g., the first patron of the day), who refused to take part in the study, who returned an empty individual questionnaire, who stated that they were under 18 years old, or who reported having food exclusions (including vegetarian or vegan diets) were excluded from the analysis. Participants who defined themselves as flexitarians were included in the study. Approval for the study was obtained from the ethics committee of Paris-Saclay University (registration number CER-Paris-Saclay-2019-016-A1).

#### Measurements

Participants’ choices were recorded by investigators using a form containing the day’s menu. A participant questionnaire was also distributed to the participants. This questionnaire collected information on participants’ age, sex, height, weight, profession, special diets, or food exclusions. This questionnaire also included questions about the social context of the meal: whether the participant knew the person who was ahead of them in the queue, whether they ate regularly with that person, and whether they felt influenced by the choices of that person. Two 7-point Likert scales were included to rate participants’ hunger before the meal and their intention to follow a vegetarian diet in the future (same question as in Study 1). Finally, participants could indicate if they refused to take part in the study.

#### Data collection procedure

Data were collected over 2 days in February–March 2020 (before any covid-19 restrictions were put in place in France) at lunch time opening between 12 pm and 2 pm. Two investigators were placed next to the cash register to record participants’ choices. A third investigator distributed individual questionnaires to participants after the cash register. At the end of the meal, the participants handed in their questionnaires to a fourth investigator who was standing at the exit of the restaurant.

#### Food choices

For a fixed price, restaurant patrons could compose a meal from three elements: one main dish and two elements from the starters and desserts on offer. The menus changed every day, but the structure of the offer remained comparable. Starters included a salad bar of fresh vegetables and about 7 ready-made starters that included mixed salads and cold meat or fish (2 to 3 vegetarian options). The main dish offer was always composed of one meat-based course, one fish-based course and one vegetarian course with one or two sides (typically, one starch and one vegetable). Desserts included a variety of dairy, fresh fruit, fruit salads and pastries.

Foods containing no meat or fish and foods containing eggs or dairy products were considered as vegetarian. Only starters and main dishes were considered for analysis because all the desserts were vegetarian according to our criteria.

#### Statistical analysis strategy

R (version 3.2.3) run with R-Studio (version 1.1.463) was used to perform statistical analyses. Binary logistic regressions were performed to test the probability of the participant making a vegetarian choice depending on whether the person ahead of them made a vegetarian choice and the participants’ dietary intention. Choices of main course and of starter were analyzed in separate models.

To study the social modeling of choices, we proceeded in two steps. First, we studied the modeling of choosing to take an item. For this step, the dependent variable was coded as 0 = “the participant did not take an item in the category of interest” (that is, a main course or starter) and 1 = “the participant took an item.” Second, we studied the modeling of choosing a vegetarian or a meat-based option. For this step, we excluded those participants who did not take a main course or a starter. The dependent variable was coded as 0 = “the participant chose a meat option” and 1 = “the participant chose a vegetarian option.” For both steps, we coded the model’s choices as 0 = “the person ahead in the queue did not take an item in the category of interest” and 1 = “the person ahead in the queue took a non-vegetarian item” and 2 = “the person ahead in the queue took a vegetarian item.” The interaction between the choices of the person ahead in the queue and the participant’s dietary intention was also included in the regression models. Regression models were adjusted for participant characteristics (age, sex, BMI, hunger score, diet) as well as for the familiarity with the person ahead of them.

### Results

#### Study population

After exclusion due to missing data and the screening criteria, the study sample included 1,037 participants (300 women, 511 men, 226 did not state their sex/gender) The average age was 29.03 years (SD = 21.65), the average BMI was 26.33 kg/m2 (SD = 2.48) (information was missing for 294 and 354 participants, respectively). Four hundred seventy-nine participants said they were omnivorous and 287 said they were flexitarian (information was missing for 255 participants). The average dietary intention was 2.5 (SD = 1.9), with 326 data points missing. Flexitarians had significantly higher vegetarian intentions compared to omnivores [3.2 (SD = 2.0) and 2.1 (SD = 1.7); T(512.96) = −8.0749, *p* < 0.0001].

#### Food choices

347 (33.5%) participants took a starter, and 690 (66.5%) participants did not take a starter. Among the participants who took a starter, 110 (32%) took a non-vegetarian starter and 237 (68%) took a vegetarian starter. The majority (978; 94%) of the participants took a main course and 59 (6%) did not take a main course. Among the participants who took a main course, 809 took a non-vegetarian option (83%) and 169 (17%) took a vegetarian option. The proportion of vegetarian choices for the main course was higher among flexitarians compared to omnivores (24 and 14%, respectively; chi-square test *p* = 0.0007).

#### Relationship with the model

Approximately half (560; 54%) of the participants stated that they knew the person ahead in the queue. Among the participants who reported knowing the person ahead, 92% also reported eating regularly with that person. Most participants reported that they did not feel that their food choices were influenced by the choices of the person ahead of them in the queue (91%).

#### Main course modeling

Binary logistic regression showed no significant interaction between the model’s choices and vegetarian intentions on the probability of taking a main course (*p* > 0.05; Table 2). Overall, participants who followed a person who took a main course had a higher chance of taking a main course compared to those who followed a person who did not take a main course. When the person ahead took a meat option, the probability of taking a main course was 377 times higher than that of not taking a main course (OR = 377.22, CI(2.5; 97.5) = [35.34;5846.54], *p* = <0.001) and it was 130 times higher when the person ahead chose a vegetarian option (OR = 130.39, CI(2.5; 97.5) = [9.89;2388.41], *p* = <0.001). The probability of taking a main course did not differ depending on whether the model chose a meat or vegetarian option (*p* = 0.320). There was no significant effect of vegetarian intentions on taking a main course (*p* = 0.48).

**Table 2:** Modeling of the main course choice and interaction with vegetarian intentions.

	OR	95%CI	*p*-Value
Taking a main course			
Model’s choice: meat-based main course	377.22	35.34–5846.54	<0.001
Model’s choice: vegetarian main course	130.39	9.89–2388.41	<0.001
Intention	1.41	0.50–3.92	0.483
Model’s choice: meat-based main course*Intention	0.98	0.31–3.44	0.974
Model’s choice: vegetarian main course*Intention	0.94	0.30–3.65	0.914
Taking a vegetarian main course			
Model’s choice: vegetarian main course	2.68	1.03–6.72	0.038
Intention	1.16	1.01–1.32	0.030
Model’s choice: vegetarian main course*Intention	0.92	0.71–1.20	0.547

#### Modeling of vegetarian main course choices

**Figure 2:**
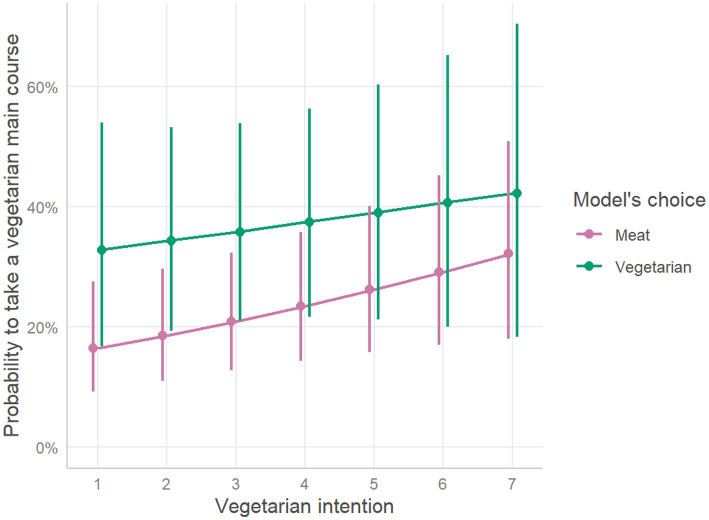
Probability to take a vegetarian main course, as function of vegetarian intention scores. The plot represents estimated effects of intentions on the probability to take a vegetarian main course for different model’s choices. Oblique lines represent the estimated marginal mean probability of taking a vegetarian main course, while the error bars represent 95% confidence intervals based on estimated standard errors (+/− 1.96 SE).

Binary logistic regression showed that participants who followed a person who chose a vegetarian main course were 2.68 times more likely to take a vegetarian main course themselves (OR = 2.68, CI(2.5; 97.5) = [1.03;6.72], *p* = 0.038). Participants with higher vegetarian intentions also were more likely to take a vegetarian main course (OR = 1.16, CI(2.5; 97.5) = [1.01;1.32], *p* = 0.030). There was no significant interaction between the model’s choice and vegetarian intentions on the likelihood of choosing a vegetarian main course (*p* = 0.547). Detailed analysis is presented in the Table 2. The likelihood of taking a vegetarian main course as a function of the model’s choice and vegetarian intentions is presented in the Figure 2.

#### Starter modeling

**Figure 3:**
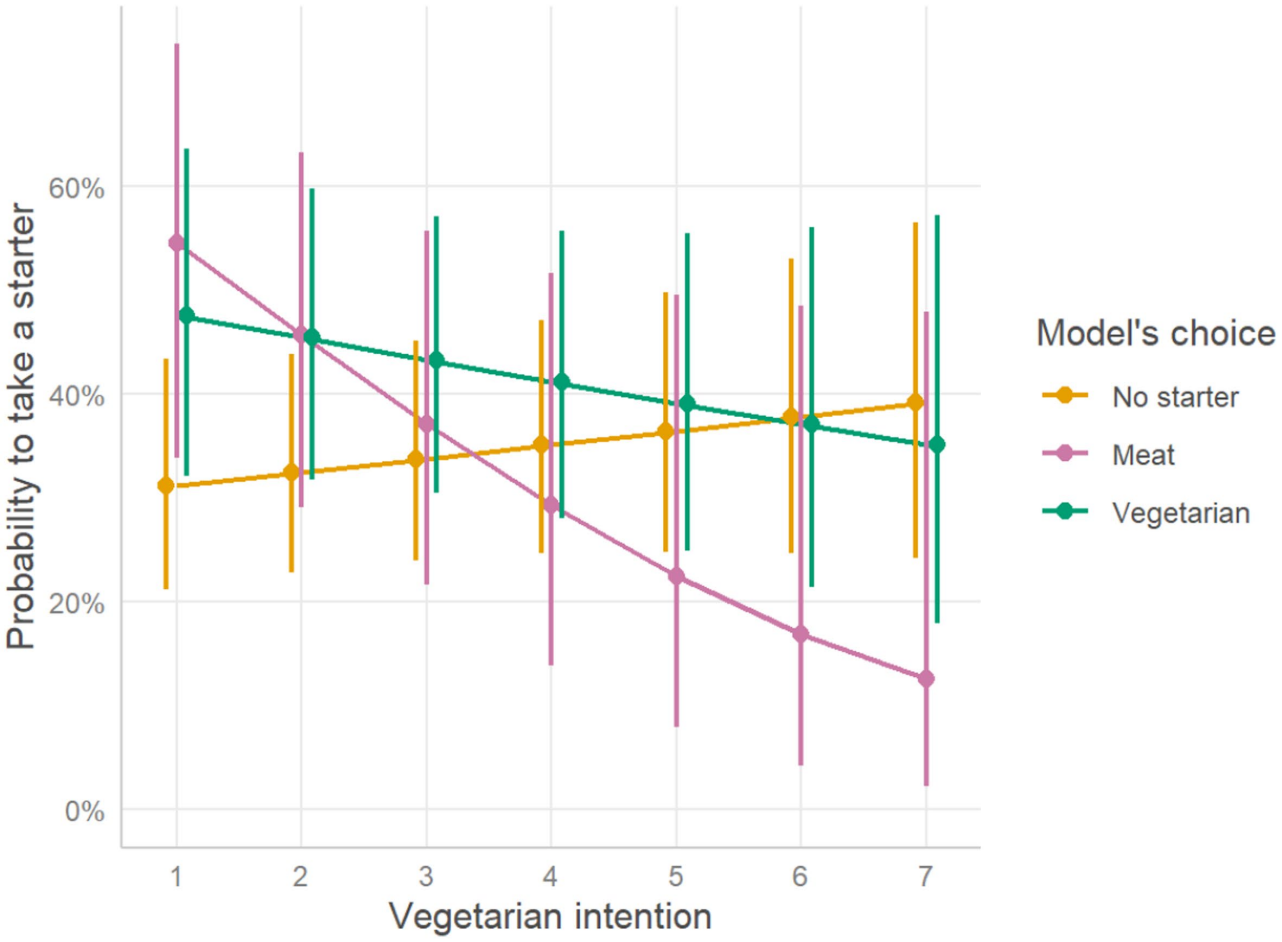
Probability to take a starter, as function of vegetarian intention scores. The plot represents estimated effects of intentions on the probability to take a starter for different model’s choices. Oblique lines represent the estimated marginal mean probability of taking a starter, as opposed to not taking a starter, while the error bars represent 95% confidence intervals based on estimated standard errors (+/− 1.96 SE).

Binary logistic regression showed a significant interaction between the model’s choice and vegetarian intentions on taking a starter: following a person who took a meat-based starter was associated with lower chances of taking a starter for participants with higher vegetarian intentions (OR = 0.66, CI(2.5; 97.5) = [0.44;0.95], *p* = 0.033) (Table 3; Figure 3). However, post-hoc tests show no difference in estimated slopes between model’s choices (*p* > 0.05). Overall, participants who followed a person who took a starter had higher chances of taking a starter compared to those who followed a person who did not take a starter, both for vegetarian (OR = 2.31, CI(2.5; 97.5) = [1.16;4.59], *p* = 0.016) and meat option (OR = 3.99, CI(2.5; 97.5) = [1.41;11.51], p = 0. 009) compared to no starter. There was no significant overall effect of vegetarian intentions on taking a starter (*p* = 0.348). See Table 3 for details.

**Table 3:** Modeling of the starter choice and interaction with vegetarian intentions.

	OR	95%CI	*P*-Value
Taking a starter			
Model’s choice: meat-based starter	3.99	1.41–11.51	0.009
Model’s choice: vegetarian starter	2.31	1.16–4.59	0.016
Intention	1.06	0.94–1.20	0.348
Model’s choice: meat-based starter*Intention	0.66	0.44–0.95	0.033
Model’s choice: vegetarian starter*Intention	0.87	0.70–1.07	0.174
Taking a vegetarian starter			
Model’s choice: meat-based starter	1.71	0.27–11.64	0.569
Model’s choice: vegetarian starter	2.89	0.85–10.44	0.095
Intention	1.54	1.16–2.13	0.005
Model’s choice: meat-based starter*Intention	0.61	0.28–1.34	0.204
Model’s choice: vegetarian starter*Intention	0.57	0.37–0.85	0.008

#### Modeling of vegetarian starter choices

**Figure 4:**
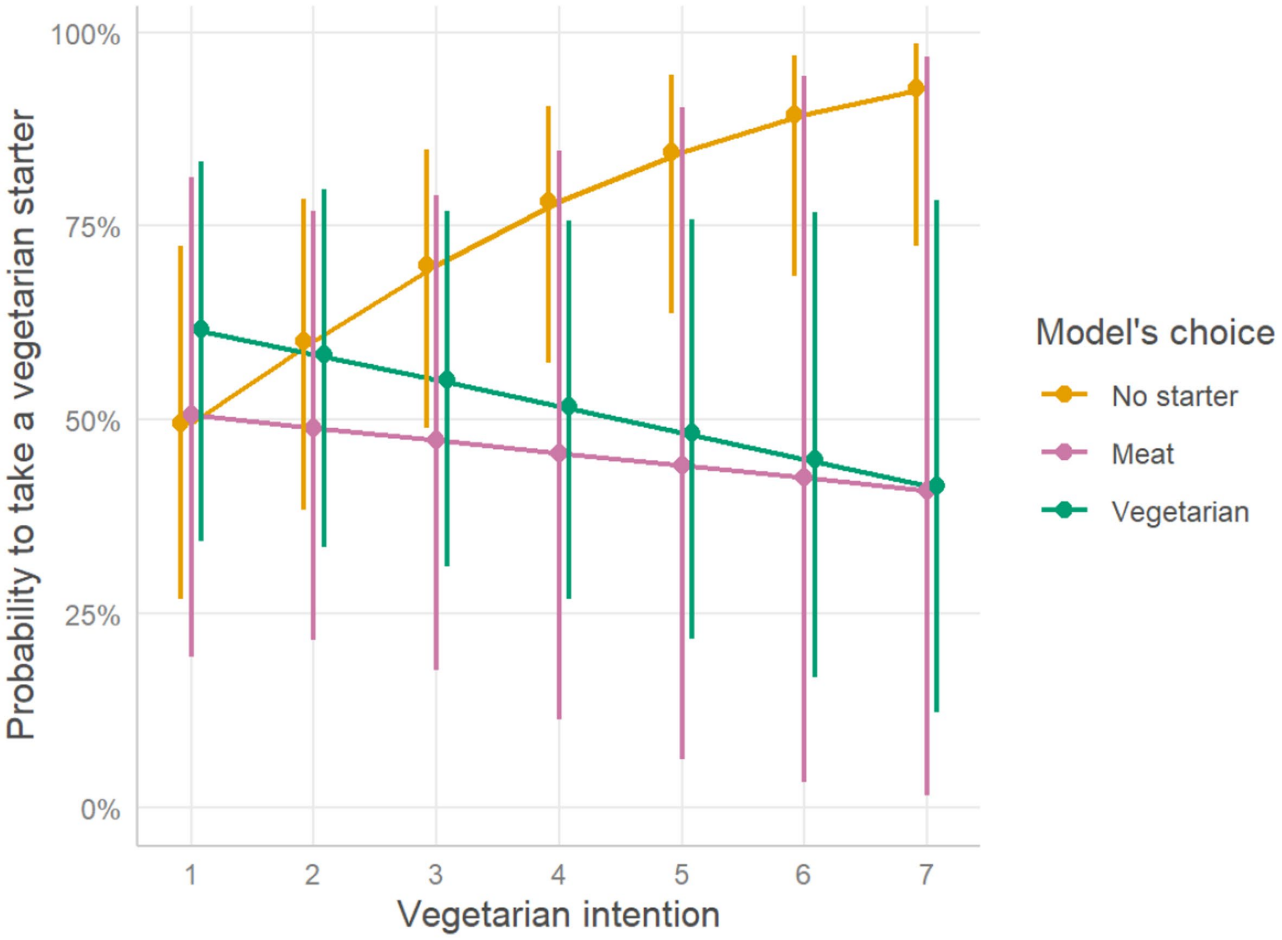
Probability to take a vegetarian starter, as function of vegetarian intention. The plot represents estimated effects of intentions on the probability to take a vegetarian starter among participants who took a starter, for different model’s choices. Oblique lines represent the estimated marginal mean probability of taking a vegetarian starter, as opposed to taking a meat starter, while the error bars represent 95% confidence intervals based on estimated standard errors (+/− 1.96 SE).

Binary logistic regression showed a significant interaction between the model’s choice and vegetarian intentions on taking a vegetarian starter: participants who followed a person who took no starter had 1.75 higher chance of taking a vegetarian starter, if their intentions to become vegetarian in the future were high, compared to participants who followed a person who took a vegetarian starter (OR = 0.57, CI(2.5; 97.5) = [0.37, 0.85], *p* = 0.008) (Table 3; Figure 4). Post-hoc tests confirm there is a difference of slopes between “vegetarian starter” and “no starter” model’s choices (Z-score = −2.86, value of *p* = 0.012). However, overall, there was no effect of the model’s choice on the chance of taking a vegetarian starter (*p* > 0.05). There was a significant positive effect of the level of vegetarian intentions on the chances of taking a vegetarian starter (OR = 1.54, CI(2.5; 97.5) = [1.16;2.13], *p* = 0.005). See Table 3 for details.

## Discussion

Across two studies we examined the moderating effect of intentions to follow a vegetarian diet on social modeling of plant-based food choices. Contrary to our predictions we did not find stronger modeling effects for individuals with low intentions to follow a vegetarian diet in the future. In fact, in Study 1, we found that participants with low intentions were *less* likely to choose plant-based food options in the presence of a vegetarian model and there was no modeling of plant-based food choices by participants with high intentions to follow a vegetarian diet. In Study 2, we found evidence for modeling of taking a plant-based main course in a restaurant but there was no moderating effect of dietary intentions. These data confirm that modeling of plant-based food choices can be observed in a naturalistic setting and that dietary intentions may moderate modeling, but further work is required to uncover the specific circumstances in which low intentions to follow a vegetarian diet might create reactance to a descriptive social norm.

In Study 1, we found that participants who had high intentions to follow a vegetarian diet in the future were not affected by the presence of the vegetarian confederate, but they chose and ate more plant-based foods overall compared with participants with low intentions. The lack of effect of the model on the choices of participants with high vegetarian intentions may be explained by the fact that they were already conforming to the social norm by choosing more of the plant-based foods. Going against our hypothesis, we also found that participants who had a low intention to become vegetarian in the future chose and ate fewer plant-based foods when eating in presence of a vegetarian confederate than when eating alone in the laboratory. We had predicted that participants with low intentions to become vegetarian might show stronger modeling for vegetarian choices due to the norm being more salient for them. However, it is possible that the circumstances of the experiment favored reactance against this norm (avoidance of plant-based foods) rather than conformity (Brehm, 1966). Indeed, the presence of a vegetarian confederate had an overall negative effect on plant-based foods intake. The reason for such reactance is unclear but theoretically might occur because participants with low intentions to become vegetarian in the future do not identify with vegetarians as a social group and may be using their food choices to distance themselves from that group (Berger and Rand, 2008). Alternatively, participants may view the declaration by the model of her vegetarianism as a threat to their ability to choose freely and so they subsequently avoided the plant-based foods as a way of regaining this freedom by rejecting the norm (Burgoon et al., 2002), especially if they perceived the model as displaying a morally superior attitude (Boenke et al., 2022). However, there are other possible explanations that should be investigated in follow-on research. For example, given that the participants with low intentions to become vegetarian only decreased their choice of plant-based options but did not increase their selection of meat options in response to the model’s choices (which might constitute a stronger form of reactance), it is possible that they were avoiding the plant-based options to be polite and to allow the vegetarian to take those options. This explanation is made less likely by the fact that the model made their choice *before* the participant and there was plenty of food on offer. Moreover, if politeness were the explanation, then this might be expected to apply to all participants and not just those with lower intentions to consume vegetarian in the future. We also note that the confederates were younger than the average age of the participants and since similarity between participants and the model can determine the extent of modeling it may have also been the case that the participant was using their food choices to dissociate themselves from the younger model (Cruwys et al., 2015). As the results of Study 1 were unexpected, it would be advisable to replicate and extend the experiment to test the underlying mechanisms. This could be achieved by including measures that could tap into the motivations underlying the food choices made by the participants, e.g., directly asking the participants *why* they made the choices they did.

In Study 2, we found evidence to support social modeling: choice of the vegetarian main meal was more likely if the person ahead in the queue choose a vegetarian meal. This finding replicates the results reported by Christie and Chen (2018) and suggests that social modeling of main dish choice in a natural setting is a robust and general phenomenon. However, there was no moderation by future dietary intentions. We did not observe any reactance to the norm (as we did in Study 1) but neither did we find that participants with low intentions to follow a vegetarian diet showed stronger modeling, which was what we had predicted. One factor that may explain this finding is that the prevailing norm was to take a meat main dish from mainly meat options (choice of 2 meat mains and 1 vegetarian option). When the usual choice is a meat course, following someone who makes a different choice (i.e., vegetarian course) may be influential because it makes the alternative more salient to everyone, including those with high intentions to become vegetarian in the future. It is possible that the reactance-like behavior observed in Study 1 was related to the manner in which the norm was conveyed and the identity of the model, which differed between studies 1 and 2. In Study 1, the norm was explicitly conveyed by the confederate who stated being vegetarian whereas in Study 2, the norm was conveyed by the implicit behavior of the person ahead in the queue. Further research is required to tease apart the potential role of explicit (versus implicit) norms and the identity of the model in how dietary intentions interact with prevailing social norms to influence choice.

The results for choice of starters were more complex than those for choice of main course in Study 2. Compared with the main course choices, there was more variability in starter choice and more available options (choice of 4–5 meat starters and 2–3 vegetarian options, plus a (vegetarian) salad bar). Most participants did not take a starter and those who did take a starter were most likely to take a vegetarian starter. This may in part explain why the modeling of starter choices that we observed was not as straightforward as for the main meals and why there were no clear modeling effects. Participants who had high intentions to become vegetarian in the future were more likely than low intention participants to choose the vegetarian starter even if the person ahead of them in the queue chose no starter, perhaps suggesting that they chose in line with their intention in the absence of a specific norm. These data further highlight that any moderating effect of intentions on of social modeling of vegetarian food choices is likely to depend on the prevailing norm and the available choices.

Across both studies we found that a higher intention to follow a vegetarian diet predicted a higher likelihood of actually choosing plant-based options regardless of the model’s choice. Intentions are not always strongly linked to actual behavior (Sheeran and Webb, 2016) but it may be that contextual aspects of the situations, e.g., presence of others and the number of vegetarian choices available served to reduce the intention-behavior gap (Carrington et al., 2010).

The results from the studies presented here should be interpreted within the light of some limitations. Given the observational design, we are unable to draw conclusions about causation from the results of Study 2. Although modeling of food choices is a plausible explanation of the data it is also possible that other factors such as similarity within social networks explains the convergence of choices. This is one reason why we conducted an experimental study alongside an observational study, to allow us to test for causality (by manipulating the norm) alongside testing whether any effect can also be observed in a natural setting (in the restaurant). As we did not observe modeling of choices in Study 1 (only reactance), further research is required to establish under what circumstances observing a model making plant-based food choices may *cause* the observer to make a similar choice (or show reactance) and how these factors might interact with dietary intentions. Well-powered laboratory-based studies could investigate the influence of factors such as the explicit versus implicit nature of the choice (e.g., drawing attention to the choice of plant-based versus choosing without commentary), the dietary identity of the model (e.g., vegetarian, flexitarian, omnivore) and similarity between the model and observer and/or perceived status as an ingroup versus outgroup member and the familiarity of the context (unfamiliar lab context versus familiar workplace canteen).

The results from these studies have implications for future research on social norm-based interventions aimed at encouraging consumers to switch to more plant-based food options. The data suggest that social norm messaging might be effective in nudging toward plant-based choices in restaurant settings and this might be the case not only for consumers already contemplating adopting a plant-based diet in the future but also for consumers who have low intentions to become vegetarian. However, care should be taken in the format of such messaging to avoid the potential for reactance. For example, it may be more effective to use messaging that avoids reference to vegetarianism or vegetarian foods in case the message is perceived to appeal only to vegetarians or to refer to a social group with which the perceiver has no affiliation or is perceived as having a morally superior attitude (Boenke et al., 2022). In addition, targeting specific food offers where the choice between meat- and plant-based is clear might also be effective.

In conclusion, the results of the present work do not provide a clear answer as to the moderating role of dietary intentions on responses to plant-based eating social norms. Rather, the data raise questions that could be addressed in follow-on studies. Specifically, the results from Study 1 suggest that participants with low intentions to follow a vegetarian diet may show norm reactance under some circumstances. Future studies could investigate whether this moderation is explained by the vegetarian identity of the model or the explicit nature of the expressed norm. The results from Study 2 suggest that the moderating role of dietary intentions may also depend upon factors such as the prevailing norm and the salience of the behavior. Although the moderation findings are not clear cut, the results of Study 2 provide further evidence of generalized social modeling of plant-based food choices in a natural setting, which could be exploited in interventions aimed at increasing more sustainable food choices.

## Data availability statement

The raw data supporting the conclusions of this article will be made available by the authors, without undue reservation.

## Ethics statement

The studies involving human participants were reviewed and approved by Ethics Committee of INSERM (CEEI) Ethics Committee of Paris-Saclay University (CER-Poléthis). The patients/participants provided their written informed consent to participate in this study.

## Author contributions

AH, ND, SH, and OD contributed to designing the studies, defining the data analysis strategy, and participated in the writing of the manuscript. AH and AG collected the study data. All authors contributed to the article and approved the submitted version.

## Funding

This research was funded by a joint PhD grant from INRAE (France) and the University of Birmingham (UK) and a research grant from AgroParisTech DRITT internal grant and by ESRC (Grant ref.: ES/P01027X/1).

## Conflict of interest

The authors declare that the research was conducted in the absence of any commercial or financial relationships that could be construed as a potential conflict of interest.

## Publisher’s note

All claims expressed in this article are solely those of the authors and do not necessarily represent those of their affiliated organizations, or those of the publisher, the editors and the reviewers. Any product that may be evaluated in this article, or claim that may be made by its manufacturer, is not guaranteed or endorsed by the publisher.
